# Passing Through a Hole: Delayed Diaphragmatic Hernia After Cytoreductive Surgery

**DOI:** 10.7759/cureus.20314

**Published:** 2021-12-09

**Authors:** Ana Mestre, André Ferreira Simões, Flávio Marino, João Gonçalves Pereira

**Affiliations:** 1 Internal Medicine, Hospital Distrital de Santarém, Santarém, PRT; 2 Intensive Care Unit Department, Hospital Vila Franca de Xira, Vila Franca de Xira, PRT; 3 Nova Medical School, Universidade Nova de Lisboa, Lisboa, PRT

**Keywords:** cytoreductive surgery, diaphragmatic hernia, peritonectomy, hypovolemic shock, advanced ovarian cancer

## Abstract

A diaphragmatic hernia is a protrusion of abdominal contents into the thoracic cavity. Although it is commonly congenital, diaphragmatic hernias can also be acquired. Blunt or penetrating trauma are among the most frequent causes, although spontaneous or iatrogenic cases have been reported. Recently, some case reports related to diaphragmatic hernia after debulking surgery for advanced ovarian cancer have been described. This is an exceedingly rare but life-threatening complication, being prompt recognition and surgical correction critical.

We report a case of a delayed diaphragmatic hernia in a 19-year-old female resulting from cytoreductive surgery for advanced ovarian cancer. Rapid evolution from gastrointestinal symptoms to hypovolemic shock occurred, and intensive care admission was required. Immediate surgery was critical to improving the patient outcome.

This case highlights this uncommon but life-threatening complication, the challenges of diagnosing and managing those patients, and the need for early recognition, support, and surgical correction.

## Introduction

Peritoneal invasion is a common feature in patients with advanced ovarian cancer, and at least two-thirds of patients with this type of cancer present with FIGO (International Federation of Gynecology and Obstetrics) stage III or IV disease at the time of diagnosis [1].

It seems that the foremost important prognostic indicator in patients with advanced-stage ovarian cancer is the volume of residual disease after surgical debulking (especially if under 1 cm); therefore, a surgical procedure with extensive cytoreduction is advised [2]. Patients should undergo a primary laparotomy with a hysterectomy and bilateral salpingo-oophorectomy. In addition, it has been recommended extensive upper abdominal surgical procedures, including omentectomy, splenectomy, distal pancreatectomy, and diaphragm peritonectomy, to attempt maximal cytoreduction and improve prognosis [3].

The most frequent complications associated with diaphragmatic peritonectomy comprise pleural effusion, pneumothorax, and pneumonia [4]. However, herniation of the abdominal contents through the diaphragm into the thoracic cavity is an uncommon but potentially fatal complication of this procedure [5].

We report a case of a diaphragmatic hernia occurring three months after cytoreductive surgery for advanced ovarian cancer.

## Case presentation

A previously healthy 19-year-old female was diagnosed with serous ovarian carcinoma, complicated with peritoneal carcinomatosis, in July 2019. By the time of August 2019, the patient underwent complete peritonectomy with a total hysterectomy and bilateral salpingo-oophorectomy. The postoperative course was uneventful. She received three courses of adjuvant chemotherapy with carboplatin over the following three months as consolidation therapy.

She was admitted to the emergency room in November 2019 with gastrointestinal complaints, including abdominal pain, vomiting, and watery diarrhea, lasting 24 hours until the admission. On admission, her physical examination was remarkable for afebrile, dehydration, hypotension (blood pressure 67/32 mmHg), and sinus tachycardia (pulse rate 120 beats/minute). An enlarged abdomen accompanied by tenderness was noted. Laboratory findings revealed elevated hemoglobin (18.6 g/dL), although with normal white blood cell and platelets count; serum urea was 73 mg/dL, creatinine 1.81 mg/dL, and this was accompanied by hypokalemia (2.96 mmol/L), normal sodium and chloride concentration, 141 mmol/L and 99 mmol/L, respectively. A C-reactive protein concentration of 2.31 mg/dL was measured; liver enzymes, total bilirubin, and lactate dehydrogenase were within the normal range. Arterial blood gas revealed metabolic acidosis and extreme hyperlactacidemia (8.95 mmol/L).

**Figure 1:**
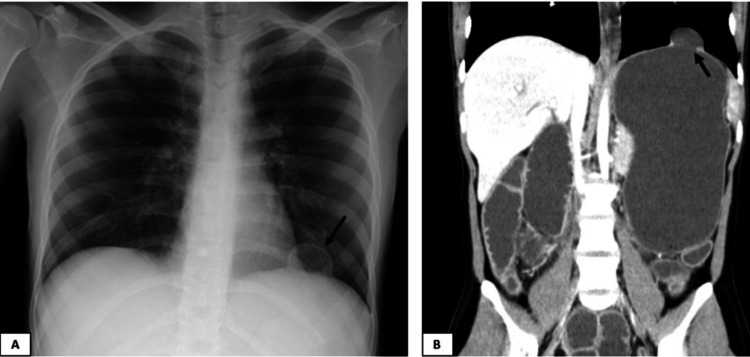
Admission chest X-ray and abdominal CT scan (A) Admission standard chest X-ray showing a small diaphragmatic hernia (arrow); (B) CT scan showing enlargement of the stomach, the bowel, and a small left diaphragmatic hernia (arrow).

**Figure 2:**
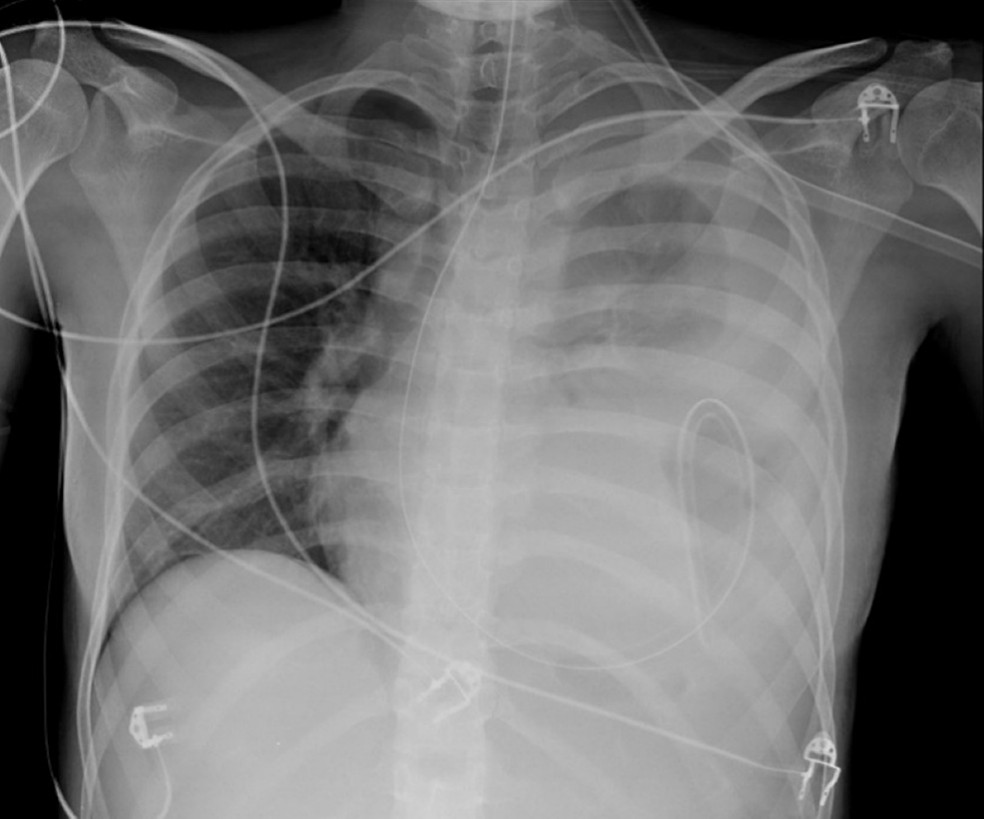
Second chest X-ray Preoperative chest X-ray showed air-fluid levels within the left hemithorax and the nasogastric tube above the diaphragm.

A standard X-ray of the abdomen was performed, showing dilatation of both small bowel and colon with scarce air-fluid levels; a standard chest X-ray was also performed, and a minor diaphragmatic defect was suspected (Figure 1A). A computerized tomography (CT) scan was carried out, confirming the enlargement of both the small and the large bowel as well as the stomach, highlighting a small diaphragmatic hernia (Figure 1B). The patient was admitted to the ICU with hypovolemic shock, which was thought to result from vomiting and diarrhea. She promptly started intravenous hydration and vasopressor support. However, she did not recover her gastrointestinal function, and sustained vomiting was noted. A second standard X-ray was performed, which in turn unveiled the stomach herniated and bulging through the thoracic cavity by means of a left diaphragmatic defect (Figure 2).

**Figure 3:**
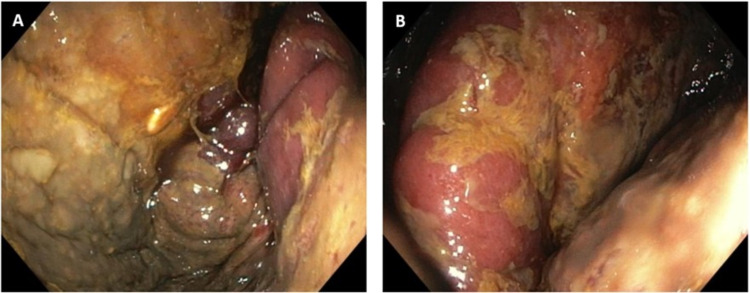
Upper endoscopy Endoscopic images revealing gastric mucosal ischemia.

Taking into account the clinical and radiological data, an upper endoscopy was urgently performed, showing signs of gastric mucosal ischemia (Figure 3). The hernial neck could not be surpassed. The patient was transferred to a tertiary hospital for urgent surgical correction by general surgery and thoracic surgery. She was submitted to a supraumbilical median laparotomy, extending to the left hypochondrium to access the diaphragmatic dome. It was a challenging procedure due to the adhesion of intestinal loops to the abdominal wall without the inner peritoneal layer. A diaphragmatic aperture of about 6 cm in diameter was recognized, which allowed the stomach to move through the thoracic cavity, building up a strangulated hernia. When opening the hernial ring, gastric repair towards the abdominal cavity was performed, acknowledging signs of ischemic suffering throughout all gastric extension, which nevertheless improved by warming maneuvers. The diaphragmatic hiatal hernia was repaired directly with a suture. Worth noticing that there were no signs of recurrence of the neoplastic disease.

**Figure 4:**
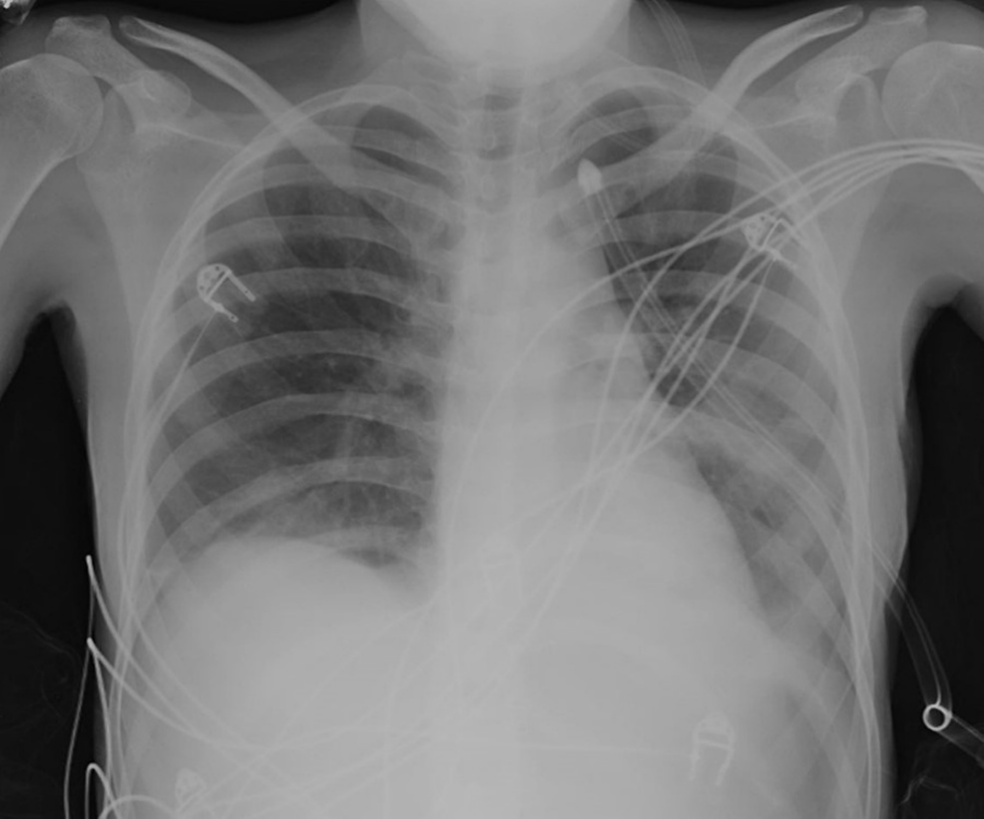
Postoperative chest X-ray Postoperative chest X-ray showing correction of the diaphragmatic hernia.

The patient presented with fever on the third postoperative day, and the chest standard X-ray showed a pleural effusion. A diagnostic thoracentesis was performed, unveiling an empyema. She was started on empirical IV antibiotics, and a pleural catheter drain was inserted. The remaining postoperative course was uneventful, with complete clinical and radiological recovery (Figure 4). She was transferred back into the incoming hospital and is now clinically disease-free.

## Discussion

The extensive upper abdominal surgical procedure, including diaphragmatic peritonectomy, is highly advised for maximal cytoreduction in patients with advanced ovarian cancer exhibiting peritoneal carcinomatosis. Diaphragmatic peritonectomy improves patient outcomes, and it is associated with a better prognosis [2], yet it is important to acknowledge that it can be quite invasive with a consequent increase in postoperative (early or delayed) complications.

A delayed diaphragmatic hernia is a rare complication after cytoreduction surgery, and very few cases have been reported in the literature [5-8]. Left-sided diaphragmatic hernia is more frequent as the liver protects the right hemidiaphragm. Resection of peritoneum and muscle tissue during diaphragm peritonectomy leads to thinning of the diaphragm and predisposes to diaphragmatic defects. Additionally, iatrogenic diaphragmatic injury during the surgery (such as thermal injury with electrocautery and accidental perforation with surgical instruments) and diaphragmatic ischemia due to peritonectomy might play a role in increasing diaphragmatic vulnerability [5,9].

Some of the reported cases of iatrogenic diaphragmatic hernias after debulking surgery have also been associated with hypothermic intraperitoneal chemotherapy (HIPEC) [6-8]; it was proposed that the cytotoxic effect of intraperitoneal chemotherapy and the upgrading of abdominal pressure during HIPEC can stretch the diaphragm resulting in the development of a hernia. Our patient, in line with other recently reported cases, was submitted to a left diaphragm peritonectomy but did not receive HIPEC [5].

Clinical presentation of diaphragmatic hernia can vary from mild and nonspecific symptoms, such as vomiting and abdominal pain, to severe gastrointestinal and respiratory symptoms with hemodynamic instability. As a result, this complication is usually misdiagnosed, and sometimes the diagnosis is overlooked until strangulation of the hernial content occurs [10]. In most reported cases, the timeline from surgery to diagnosis of diaphragmatic hernia appears to be several weeks to months [5]. In our case, the patient presented with acute obstructive disease and severe hemodynamic compromise three months after the cytoreductive surgery, and emergency surgery was required.

Computed tomography is considered the gold standard technique to establish the diagnosis, allowing the evaluation of the size, location, and content of the diaphragmatic defect [11]. Despite its limited findings, standard chest X-ray is a reasonable screening examination, in which the most prevailing radiologic finding is the opaqueness of the hemithorax, with dilated intestinal loops and air-fluid levels within the thorax [12]. Furthermore, the nasogastric tube position above the diaphragm should raise the suspicion of an abdominal organ in the thoracic cavity, as seen in our patient’s standard chest X-ray.

The treatment of choice for diaphragmatic hernia is surgery. Patients with a symptomatic hernia should undergo urgent surgery, while in case of asymptomatic diaphragmatic hernia the repair procedure may be elective. Regardless of the size of the defect, all diagnosed diaphragmatic hernias require surgical repair to avoid serious complications such as incarceration, strangulation, and rupture [11]. In complex situations, as in the above case, it is essential to provide adequate support with fluids and electrolytes to compensate for the extreme dehydration secondary to gastrointestinal ischemia. No specific measures have been demonstrated to prevent this unusual complication. The integrity of the diaphragm should be carefully assessed intraoperatively, and every defect should be closed to prevent the possible development of a hernia.

## Conclusions

We report a case of a delayed diaphragmatic hernia after cytoreductive surgery with diaphragmatic peritonectomy for advanced ovarian cancer. This is a rare complication, challenging to predict and diagnose. Reviewing the patient’s surgical history and recognizing the symptoms are crucial to early detection of this potentially life-threatening complication. Surgical repair is the treatment of choice for iatrogenic diaphragmatic hernia.
